# Prediction of the toxic effects of (agro) chemical mixtures on organisms using simple time-based models

**DOI:** 10.1016/j.mex.2022.101956

**Published:** 2022-12-02

**Authors:** Kingsley Chukwuemeka Kanu

**Affiliations:** Department of Environmental Management and Toxicology, Michael Okpara University of Agriculture, Umudike, Nigeria

**Keywords:** Lethal time, Predictive model, Pesticide mixture, Acute toxicity

## Abstract

The lethal effect of a chemical acting alone can be predicted using the simple hyperbolic model, which relies on the chemicals' median lethal time (LT_50_). However, this model cannot be used to predict mixture toxicity, considering that toxicity in natural ecosystems often results from exposure to mixtures rather than single chemicals. The lethal time addition method was developed to calculate the LT_50_ of a pesticide mixture from the LT_50_ of its components. It enables the hyperbolic model to estimate the lethal effects of a mix of pesticides at various exposure times. The hyperbolic model, complemented by the lethal-time addition model, predicted the percentage mortality of *Clarias gariepinus* and *Oreochromis niloticus* exposed to binary and quaternary mixtures of atrazine, mancozeb, chlorpyrifos, and lambda-cyhalothrin and estimated the 96 hr LC_50_ of the pesticide mixture.

## Highlights

• The lethal time addition (LT) model was used to calculate the LT_50_ of a chemical mixture.
• With the LT model, the hyperbolic model could be used to predict chemical mixture toxicity.
• Together both models may be helpful for the risk assessment of chemical mixtures, including pesticides.

## Graphical abstract

**Figure:**
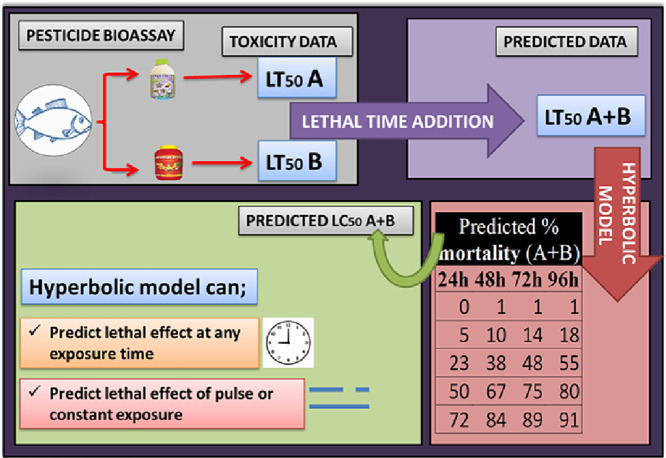

Specifications Table

**Table:** 

Subject area	Environmental Science
More specific subject area	Prediction of toxicity of chemical mixtures
Method name	An approach for calculating the median lethal time (LT_50_) values for a chemical mixture for use in the hyperbolic model
Name and reference of the original method	The lethal time addition model is novel
Resource availability	Not applicable

## Introduction

Pollutants exist as a mixture in the aquatic environment. The main goal of ecotoxicology is to predict the toxic effects of contaminants in the environment [1,2]. While animal models like fish are commonly used in the laboratory to predict toxicity by studying their biological responses to toxicants, there are increasing calls for alternative techniques like statistical or mathematical models that do not necessitate the use of animals [3]. Besides, it is also not feasible to perform toxicity tests for a mixture to assess joint toxicity due to many pollutants.

Previous authors have shown that a hyperbolic model which utilises the Michaelis-Menten mathematical expression could adequately describe the lethality of a pollutant over time [4]. The hyperbolic model only depends on two variables: the toxicant concentration and the exposure time. It is a straightforward model that permits the prediction of mortality for any combination of concentration, and time, whether they result from pulsed, post-acute, or continuous exposure to toxicants. The median lethal time (LT_50_) is a vital component of the hyperbolic model, which must be determined for each toxicant concentration. It can be estimated using probit, logit, or Weibull models in a time-to-death bioassay. Toxic effects for concentrations other than those tested could also be evaluated using a complementary log-to-log model to calculate all LT_50_ values for a toxicant [1].

A limitation of the hyperbolic model is that it is only used to predict mortality for single chemicals. It cannot predict the toxicity of a mixture of pollutants. This limitation is significant because toxicity in natural ecosystems often results from exposure to mixtures of toxicants rather than a single toxicant [5,6]. Pesticides are one such class of pollutants that co-exists in the environment. The aquatic ecosystem is a sink for pesticides [7] which enter the aquatic ecosystems through direct application, spray drift, surface runoff from soil/pavement, depositions (both dry and rainy), and urban/industrial discharges [8].

Can the hyperbolic model predict the toxic effects of mixtures of toxicants/pesticides? The answer probably lies in exploring ways of computing the LT_50_ of the mix of toxicants/pesticides from the LT_50_ of the individual toxicants/pesticides in the combination. This study presents a method for calculating the LT_50_ of binary and quaternary toxicant mixtures from the LT_50_ of the individual toxicant components that make up the mixture. The estimated LT_50_ of the mix can then be used in the hyperbolic model to predict the mortality of organisms at any time of exposure. Alternatively, the derived mixture LT_50_ can be used with other models since they represent the median effect values for specific concentrations.

## Methods

### Study design

**Fig. 1:**
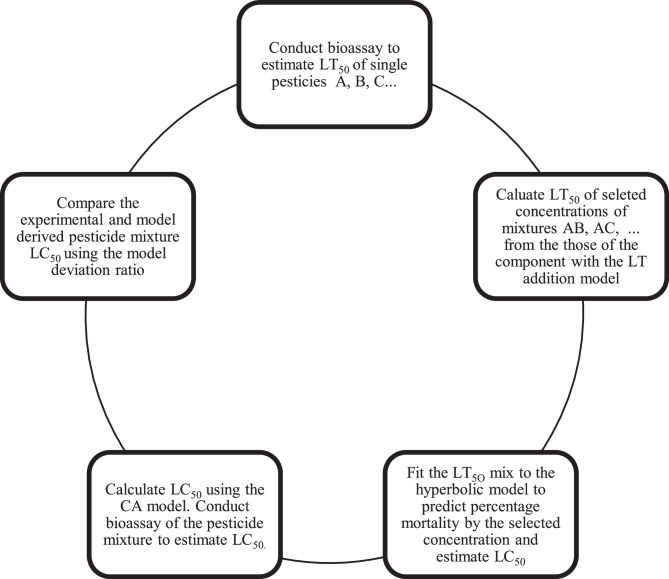
**Overview of the study design**.

Fig. 1 shows an overview of the tasks in the study.

#### Bioassay of single pesticides

The primary data is the median lethal times (LT_50_s) of single pesticides, atrazine, mancozeb, chlorpyrifos, and lambda-cyhalothrin, obtained from a 96-hour acute toxicity study. Some of this data was published in an earlier study [9]. The LT_50_ data and the corresponding concentration were fitted to a regression model to get the regression coefficients *a* and *b* so that the LT_50_ of concentrations beyond those tested experimentally could be obtained.

Standard procedures were followed to perform the toxicity tests [10]. The range-finding test was used to establish the concentrations for the actual toxicity test. Ten *C. gariepinus* and *O. niloticus* fingerlings were exposed in duplicates to different pesticide concentrations in 4-litre plastic aquaria containing one litre of the test solutions. Mortality count and time-to-death were recorded every hour, while the LC_50_ and LT_50_ were estimated at the end of 96 h.

#### Calculation of pesticide mixture LT_50_ using the lethal time addition model

A range of binary and quaternary pesticide mixture concentrations were selected, considering the toxicity (concentration range from the acute toxicity test) of the major component in the mixture. The concentration range included concentrations expected to cause below and above 50% mortality. The concentration of each element in the pesticide mixture was then calculated based on their ratio. The LT_50_ of the individual pesticide concentration in each mixture was then estimated using the regression equation In (LT_50_) = *a* + *b.*In (*C*) [1], where *a* and *b* are the regression coefficients obtained from the fitted acute toxicity data of the single pesticides.

Once the LT_50_ of the individual pesticides in the mixture was estimated, the lethal time model Eq. (1) was used to calculate the LT_50_ of the pesticide mixtures.

Eq. (1) shows the complimentary lethal time addition model

(1) $$LT50\;\;mix=\frac{1}{\frac{Ca}{LTa}+\frac{Cb}{LTb}+\ldots }$$

Ca and Cb are the concentration of each pesticide (a and b) in the mixture, and LT _a_ and LT_b_ are the lethal time of the concentrations of the pesticides a and b

#### Prediction of pesticide mixture mortality using the hyperbolic model

The calculated pesticide mixture LT_50_ was subsequently fitted into the hyperbolic model (Eq. (2) to predict the percentage mortality of the mixture at different exposure duration. However, only the 96 hr LC_50_ of the mixtures were subsequently estimated.

Eq. (2) shows the hyperbolic model [4] used to predict the percentage mortality.

(2) $$P=P100\times t/\left(LT_{50}+t\right)$$

*P* = percentage of the animals that would die at different times (t), *t* = duration, e.g., 24, 48, 72, and 96 h. P100 = total mortality of the population (100%).

#### Calculation of pesticide mixture median lethal concentration using concentration addition model

The 96 hr LC_50_ of the pesticide mixtures were also estimated using the concentration addition (CA) model [11] (Eq. (3)), A range of percentage mortality was selected (5, 15, 35, 60, and 90%), and the lethal concentration LCx1 and LCx2 of the single pesticides that would produce this percentage mortality were identified from the probit analysis of the single pesticides. They were then combined using the CA model to get the lethal concentration of the mixture.

(3) $$LCx\;mix=\frac{LCx1}{P1}+\frac{LCx2}{P2}\;$$

LCx mix is the lethal concentration of the mixture, while LCx1 and LCx2 are the lethal concentrations of the single pesticides 1 and 2 that would produce the same percentage of mortality x. P1 and P2 are the proportion of each pesticide 1 and 2 in the mixture.

#### Bioassay of binary and quaternary pesticide mixture

Acute toxicity test of the pesticide mixtures was performed. Pesticide mixtures were prepared based on each fish species' equitoxic pesticide ratio (ratio of the 96-hr LC50 of single pesticides) [12]. The ratio of the pesticides in the mixture and the stock solution is outlined below;

**Table:** 

Pesticide mixture	*C. gariepinus*	*O. niloticus*
	Mixture ratio	Mixture ratio
Atrazine: Mancozeb	1:1.39 (2.39 g/L)	3.61:1 (4.61 g/L)
Atrazine: Chlorpyrifos	33.95:1 (3.495 g/L)	98.47:1 (9.84 g/L)
Mancozeb: Chlorpyrifos	47.35:1 (4.84 g/L)	27.3:1 (2.83 g/L)
Atrazine: Lambda cyhalothrin	40.28:0.001(4.0301 g/L)	12.99:0.01 (13 g/L)
Mancozeb: Lambda cyhalothrin	56.18:0.001 (5.6201 g/L)	3.60:0.01 (3.61 g/L)
Lambda cyhalothrin: Chlorpyrifos	0.001:1.18 (0.1188 g/L)	1:13.21 (0.355 g/L)
Quaternary mixture (A:M:C:L)	1.299:0.360:0.0132:0.001 (9.7651 g/L)	1.299:0.360:0.0132:0.001 (16.732 g/L)

AMCL-atrazine, mancozeb, chlorpyrifos, and lambda-cyhalothrin; values in bracket are the stock solution.

Stock solutions were diluted to prepare the test solutions of different concentrations. The same procedure described for studying the acute toxicity of single pesticides was followed to study the acute toxicity of binary and quaternary mixtures. The 96-hr LC_50_ of the mixtures was estimated at the end of 96 h. The fingerlings' mean length and weight were 2.91±0.07 cm and 0.22±0.01 g, respectively, for *C. gariepinus*, 3.69±0.11 cm, and 0.45±0.04 g, respectively for *O. niloticus*.

### Data analyses

The LT_50_s and LC_50_s of single pesticides and LC_50_s of the pesticide mixtures from the bioassay were estimated using probit analysis. Probit analysis and linear regression were computed with SPSS version 25. For the linear regression, the dependent variable (y) was the LT_50,_ and the independent variable (x) was the external chemical concentration C. The model deviation ratio (MDR) was used as a quantitative measure for the compliance between observed mixture toxicity and the toxicity predicted by the hyperbolic model and the concentration addition model [13]. The valid MDR values range from 0.5 to 2.

$$\text{MDR}=\frac{\mathrm{Predicted}\;\mathrm{LCx}\;\mathrm{mix}}{\mathrm{Observed}\;\mathrm{LCx}\;\mathrm{mix}}$$

## Results

### Bioassay of single pesticides

The 96-hour LC_50_ of each pesticide and LT_50_ of the concentrations used for the single pesticides are presented in Table 1. At 96 h, not up to 50% mortality had occurred for the concentrations marked with asterisks; thus, the LT_50_ > 96 h was extrapolated from the probit analysis.

**Table 1:** Estimated LT_50_ for *C. gariepinus* and *O. niloticus*.

Pesticides	Catfish			Nile tilapia		
	Concentration (mg/l)	96hr LC_50_ (95% C.I) (mg/L)	LT_50_ (h)	Concentration (mg/l)	96hr LC_50_ (95% C.I) (mg/L)	LT_50_ (h)
Atrazine	13*	17.463 (15.486–19.201)	148.82	6*	10.926 (8.850–13.409)	253.92
	16*		149.70	9*		156.13
	19*		103.85	14		58.11
	21		53.55	21		27.49
	24		16.79			
Chlorpyrifos	0.25*	0.515 (0.349–0.786)	538.44	0.08*	0.111 (0.103–0.121)	646.81
	0.4*		209.83	0.10*		245.68
	0.55		49.59	0.13		22.02
	0.7		29.85	0.17		10.19
	1		8.28			
Mancozeb	8*	24.383 (16.360–31.253)	1115.47	1.4*	3.028 (2.665–3.424)	2769.46
	20*		226.26	2.2*		500.02
	32		80.56	3.2		58.07
	38		39.66	4.8		13.56
	42		13.01			
Lambda-cyhalothrin	0.00025*	0.434 (0.384–0.482)^+^	214.82	0.0025*	8.412 (7.015–10.062^+^	317.13
	0.00038*		103.90	0.0050*		221.85
	0.00050		37.17	0.0075*		110.75
	0.00063		10.40	0.0100		42.61
	0.00075		3.24	0.0125		28.52
				0.0150		15.20

+ µg/L.

Table 2 shows the parameters for the regression equation In(LT_50_) = *a* + *b.*In(*C*) obtained by linear regression on the natural logarithm transformed data of both y (lethal time) and x (pesticide concentration) variables (Table 1). A good fit to the model was obtained, with r^2^ values above 0.75 in all cases.

**Table 2:** Regression parameters obtained from fitting the data in Table 1 in a linear regression model.

Species	Pesticide	Intercept (*a*)	Slope (*b*)	r^2^	N
Catfish	Atrazine	14.033	−3.358	0.758	5
	Chlorpyrifos	2.211	−3.069	0.984	5
	Mancozeb	12.244	−2.397	0.892	5
	Lambda-cyhalothrin	−25.581	−3.784	0.915	5
Nile tilapia	Atrazine	8.906	−1.826	0.982	4
	Chlorpyrifos	−8.373	−5.889	0.939	4
	Mancozeb	9.482	−4.439	0.988	4
	Lambda-cyhalothrin	−4.161	−1.725	0.884	6

N (number of points in the regression) represents the number of concentrations used in the bioassay.

### Calculation of the LT_50_ of binary and quaternary pesticide mixture

The calculated LT_50_ of each selected concentration of the binary and quaternary pesticide mixture is presented in Tables 3 and 4 for *C. gariepinus* and *O. niloticus,* respectively.

**Table 3:** Calculated LT_50_ of binary and quaternary pesticide mixture for *C. gariepinus*.

Pesticide Mixture	Sel. conc (mg/l)	Ca		Cb		LT_50_ a		LT_50_ b		Calculated LT_50_ mix
Atrazine-Mancozeb	5	2.1		2.9		35,407		34,493		34,869.6
	10	4.2		5.8		6722		3364		425.3
	15	6.3		8.7		2543		862		79.4
	20	8.4		11.6		1276		328		23.8
	25	10.5		14.5		748		155		9.3
Atrazine-Chlorpyrifos	4	3.9		0.1		13,034		7069		3181.64
	8	7.8		0.2		1271		842		156.62
	12	11.7		0.3		326		243		26.88
	16	15.5		0.5		124		100		7.70
	20	19.4		0.6		59		51		2.92
Atrazine-Lambda cyhalothrin	3.5	3.49991		0.00009		18,514		18,088		5289.83
	7.5	7.49981		0.00019		1432		1011		190.97
	11.5	11.49971		0.00029		341		201		29.65
	15.5	15.49962		0.00038		125		65		8.07
	19.5	19.49952		0.00048		58		27		2.97
Mancozeb-Chlorpyrifos	1.5	1.47		0.03		82,636		388,342		55,090.41
	4.5	4.41		0.09		5936		13,333		1319.15
	7.5	7.34		0.16		1745		2780		232.63
	10.5	10.28		0.22		779		990		74.18
	15.5	15.18		0.32		306		300		19.76
Mancozeb-Lambda cyhalothrin	4	3.99993		0.00007		7488		38,426		1872.10
	8	7.99986		0.00014		1422		2789		177.72
	12	11.99979		0.00021		538		601		44.83
	16	15.99972		0.00028		270		203		16.87
	20	19.99964		0.00036		158		87		7.91
Lambda cyhalothrin-Chlorpyrifos	0.05	0.04996		0.00004		89,993.04		280,551.2267		1,800,890.66
	0.25	0.24979		0.00021		644.273		6.3713E+279		2579.26
	0.45	0.44962		0.00038		106.0813		1.17626E+46		235.93
	0.65	0.64945		0.00055		34.31759		8.01565E+14		52.84
	0.85	0.84928		0.00072		15.06472		3,487,596.393		17.74
Quaternary Mixture	**Sel. conc (mg/l)**	**Ca**	**Cb**	**Cc**	**Cd**	**LT_50_ a**	**LT_50_ b**	**LT_50_ c**	**LT_50_ d**	**Calculated LT_50_ mix**
	8	3.3001	4.60	0.0972	0.0001	22,554	5349	11,666	22,594	985.14
	12	4.9501	6.90	0.1458	0.0001	5780	2024	3361	4871	231.97
	16	6.6003	9.20	0.1944	0.0001	2200	1016	1390	1640	81.94
	20	8.2503	11.50	0.2430	0.0002	1040	595	701	705	36.20
	24	9.9003	13.80	0.2916	0.0003	564	384	401	354	18.44

Sel. Conc- selected concentration.

**Table 4:** Calculated LT_50_ of binary and quaternary pesticide mixture for *O. niloticus*.

Pesticide Mixture	Sel. conc (mg/l)	Ca		Cb		LT_50_ a		LT_50_ b		Calculated LT_50_ mix
Atrazine-Mancozeb	1.5	1.2		0.3		5498		1,916,371		4676.83
	5	3.9		1.1		610		9150		153.00
	8.5	6.7		1.8		232		868		32.39
	12	9.4		2.6		123		188		11.11
	15.5	12.1		3.4		77		60		4.70
Atrazine-Chlorpyrifos	3	2.97		0.03		1011		207,618		340.30
	5	4.95		0.05		398		10,252		80.31
	7	6.93		0.07		215		1413		31.00
	9	8.91		0.09		136		322		15.19
	11	10.89		0.11		94		99		8.57
Atrazine-Lambda cyhalothrin	2.5	2.498		0.002		1254		755		501.43
	5	4.996		0.004		354		228		70.72
	7.5	7.494		0.006		169		113		22.49
	10	9.992		0.008		100		69		9.97
	12.5	12.490		0.010		66		47		5.31
Mancozeb-Chlorpyrifos	1.5	1.45		0.05		2545		7520		1696.58
	2.25	2.17		0.08		421		691		186.99
	3	2.89		0.11		117		127		39.11
	3.75	3.62		0.13		44		34		11.62
	4.5	4.34		0.16		19		12		4.31
Mancozeb-Lambda cyhalothrin	1.3	1.296		0.004		4145		256		3059.71
	2.1	2.094		0.006		493		112		232.65
	2.9	2.892		0.008		118		64		40.49
	3.7	3.690		0.010		40		42		10.79
	4.5	4.488		0.012		17		30		3.72
Lambda cyhalothrin-Chlorpyrifos	0.12	0.112		0.008		94		59		751.57
	0.15	0.139		0.011		25		40		172.88
	0.18	0.167		0.013		9		29		50.46
	0.21	0.195		0.015		3		22		17.63
	0.24	0.223		0.017		2		18		7.06
Quaternary Mixture	**Sel. conc (mg/l)**	**Ca**	**Cb**	**Cc**	**Cd**	**LT_50_ a**	**LT_50_ b**	**LT_50_ c**	**LT_50_ d**	**Calculated LT_50_ mix**
	3	2.33	0.65	0.02	0.002	1575	91,610	867,558	852	672.17
	5.5	4.27	1.18	0.04	0.003	521	6215	24,438	299	119.02
	8	6.21	1.72	0.06	0.005	263	1178	2690	157	39.76
	10.5	8.15	2.26	0.08	0.006	160	352	542	98	17.36
	13	10.09	2.80	0.10	0.008	108	136	154	68	8.73

Sel. Conc- selected concentration.

### Prediction of the percentage mortality using the hyperbolic model

Tables 5 and 6 show the predicted percentage mortality for *C. gariepinus* and *O. niloticus,* respectively, for the selected concentration at different exposure times.

**Table 5:** Predicted percentage mortality for *C. gariepinus*.

Pesticide Mixture	Selected concentration (mg/l)	Calculated LT_50_ mix (hours)	Predicted percentage mortality			
			24 h	48h	72h	96h
Atrazine-Mancozeb	5	6973.9	0	1	1	1
	10	425.3	5	10	14	18
	15	79.4	23	38	48	55
	20	23.8	50	67	75	80
	25	9.3	72	84	89	91
Atrazine-Chlorpyrifos	4	3181.64	1	1	2	3
	8	156.62	13	23	31	38
	12	26.88	47	64	73	78
	16	7.70	76	86	90	93
	20	2.92	89	94	96	97
Atrazine-Lambda cyhalothrin	3.5	5289.83	0	1	1	3
	7.5	190.97	11	20	27	33
	11.5	29.65	45	62	71	76
	15.5	8.07	75	86	90	92
	19.5	2.97	89	94	96	97
Mancozeb-Chlorpyrifos	1.5	55,090.41	0	0	0	0
	4.5	1319.15	2	4	5	7
	7.5	232.63	9	17	24	29
	10.5	74.18	24	39	49	56
	15.5	19.76	55	60	70	75
Mancozeb-Lambda cyhalothrin	4	1872.10	1	2	4	5
	8	177.72	12	21	29	35
	12	44.83	35	52	62	68
	16	16.87	59	74	81	85
	20	7.91	75	86	90	92
Lambda cyhalothrin-Chlorpyrifos	0.05	1,800,890.66	0	0	0	0
	0.25	2579.26	1	2	3	4
	0.45	235.93	9	17	23	29
	0.65	52.84	31	48	58	64
	0.85	17.74	58	73	80	84
Quaternary Mixture	8	985.14	2	5	7	9
	12	231.97	9	17	24	29
	16	81.94	23	37	47	54
	20	36.20	40	57	67	73
	24	18.44	57	72	80	84

**Table 6:** Predicted percentage mortality for *O.niloticus*.

Pesticide Mixture	Selected concentration (mg/l)	Calculated LT_50_ mix (hours)	Predicted percentage mortality			
			24 h	48h	72h	96h
Atrazine-Mancozeb	1.5	4676.83	1	1	2	2
	5	153.00	14	24	32	39
	8.5	32.39	43	60	69	75
	12	11.11	68	81	87	90
	15.5	4.70	84	91	94	95
Atrazine-Chlorpyrifos	3	340.30	7	12	17	22
	5	80.31	23	37	47	54
	7	31.00	44	61	70	76
	9	15.19	61	76	83	86
	11	8.57	74	85	89	92
Atrazine-Lambda cyhalothrin	2.5	501.43	5	9	13	16
	5	70.72	25	40	50	58
	7.5	22.49	52	68	76	81
	10	9.97	71	83	88	91
	12.5	5.31	82	90	93	95
Mancozeb-Chlorpyrifos	1.5	1696.58	1	3	4	5
	2.25	186.99	11	20	28	34
	3	39.11	38	55	65	71
	3.75	11.62	67	81	86	89
	4.5	4.31	85	92	94	96
Mancozeb-Lambda cyhalothrin	1.3	3059.71	1	2	2	3
	2.1	232.65	9	17	24	29
	2.9	40.49	37	54	64	70
	3.7	10.79	69	82	87	90
	4.5	3.72	87	93	95	96
Lambda cyhalothrin-Chlorpyrifos	0.12	751.57	3	6	9	11
	0.15	172.88	12	22	29	36
	0.18	50.46	32	49	59	66
	0.21	17.63	58	73	80	84
	0.24	7.06	77	87	91	93
Quaternary Mixture	3	672.17	3	7	10	12
	5.5	119.02	17	29	38	45
	8	39.76	38	55	64	71
	10.5	17.36	58	73	81	85
	13	8.73	73	85	89	92

### Prediction of pesticide mixture toxicity using concentration addition model

The calculated lethal concentration of the mixtures (LCmix) for *C. gariepinus* and *O. niloticus,* respectively, that would cause the selected percentage mortality, is presented in Tables 7 and 8.

**Table 7:** Calculated lethal concentration of the mixtures (LC mix) for *C. gariepinus*.

Pesticide mixture	Selected% mortality	LCx1		LCx2		P1		P2		Cal. LCmix
Atrazine-Mancoze	5%	7.025		5.363		0.58		0.42		6.22
	15%	10.894		12.398		0.58		0.42		11.48
	35%	15.033		19.928		0.58		0.42		16.76
	60%	19.094		27.313		0.58		0.42		21.84
	90%	25.631		39.203		0.58		0.42		29.97
Atrazine-Chlorpyrifos	5%	7.025		.125		0.97		0.03		2.73
	15%	10.894		.270		0.97		0.03		5.12
	35%	15.033		.424		0.97		0.03		7.57
	60%	19.094		.575		0.97		0.03		9.94
	90%	25.631		.819		0.97		0.03		13.73
Atrazine-Lambda cyhalothri	5%	7.025		0.0002		0.99998		0.00002		3.62
	15%	10.894		0.0003		0.99998		0.00002		5.52
	35%	15.033		0.0004		0.99998		0.00002		7.55
	60%	19.094		0.0005		0.99998		0.00002		9.53
	90%	25.631		0.0006		0.99998		0.00002		12.73
Mancozeb-Chlorpyrifos	5%	5.363		.125		0.98		0.02		2.88
	15%	12.398		.270		0.98		0.02		6.42
	35%	19.928		.424		0.98		0.02		10.21
	60%	27.313		.575		0.98		0.02		13.93
	90%	39.203		.819		0.98		0.02		19.91
Mancozeb-Lambda cyhalothrin	5%	5.363		0.000186		0.99998		0.00002		3.54
	15%	12.398		0.000278		0.99998		0.00002		6.91
	35%	19.928		0.000376		0.99998		0.00002		10.26
	60%	27.313		0.000473		0.99998		0.00002		13.47
	90%	39.203		0.000628		0.99998		0.00002		18.57
Lambda cyhalothrinChlorpyrifos	5%	0.125		0.000186		0.999		0.001		0.08
	15%	0.270		0.000278		0.999		0.001		0.15
	35%	0.424		0.000376		0.999		0.001		0.22
	60%	0.575		0.000473		0.999		0.001		0.28
	90%	0.819		0.000628		0.999		0.001		0.39
Quaternary Mixture		LCx1	LCx2	LCx3	LCx4	P1	P2	P3	P4	Cal. LCmix
	5%	7.025	5.363	0.125	0.0002	0.413	0.575	0.012	0.00001	3.14
	15%	10.893	12.398	0.269	0.0003	0.413	0.575	0.012	0.00001	6.02
	35%	15.033	19.928	0.424	0.0004	0.413	0.575	0.012	0.00001	8.91
	60%	19.094	27.313	0.575	0.0005	0.413	0.575	0.012	0.00001	11.70
	90%	25.631	39.203	0.819	0.0006	0.413	0.575	0.012	0.00001	16.15

**Table 8:** Calculated lethal concentration of the mixtures (LCmix) for *O. niloticus*.

Pesticide mixture	Selected% mortality	LCx1		LCx2		P1		P2		Cal. LCmix
Atrazine-Mancozeb	5%	3.95		1.87		0.78		0.22		3.18
	15%	5.76		2.23		0.78		0.22		4.29
	35%	8.61		2.70		0.78		0.22		5.84
	60%	12.78		3.26		0.78		0.22		7.83
	90%	24.14		4.42		0.78		0.22		12.26
Atrazine-Chlorpyrifos	5%	3.95		0.08		0.99		0.01		2.64
	15%	5.76		0.09		0.99		0.01		3.51
	35%	8.61		0.10		0.99		0.01		4.69
	60%	12.78		0.12		0.99		0.01		6.12
	90%	24.13		0.15		0.99		0.01		9.08
Atrazine-Lambda cyhalothrin	5%	3.95		0.003		0.9992		0.0008		1.91
	15%	5.76		0.004		0.9992		0.0008		2.81
	35%	8.61		0.007		0.9992		0.0008		4.27
	60%	12.78		0.010		0.9992		0.0008		6.43
	90%	24.13		0.020		0.9992		0.0008		12.42
Mancozeb-Chlorpyrifos	5%	1.87		0.08		0.96		0.04		1.03
	15%	2.23		0.09		0.96		0.04		1.21
	35%	2.70		0.10		0.96		0.04		1.42
	60%	3.26		0.12		0.96		0.04		1.67
	90%	4.42		0.15		0.96		0.04		2.17
Mancozeb-Lambda cyhalothrin	5%	1.87		0.003		0.997		0.003		0.66
	15%	2.23		0.004		0.997		0.003		0.91
	35%	2.70		0.007		0.997		0.003		1.26
	60%	3.26		0.010		0.997		0.003		1.71
	90%	4.42		0.020		0.997		0.003		2.73
Lambda cyhalothrin-Chlorpyrifos	5%	0.08		0.003		0.93		0.07		0.03
	15%	0.09		0.004		0.93		0.07		0.04
	35%	0.10		0.007		0.93		0.07		0.05
	60%	0.12		0.011		0.93		0.07		0.07
	90%	0.15		0.023		0.93		0.07		0.11
Quaternary Mixture		LCx1	LCx2	LCx3	LCx4	P1	P2	P3	P4	Cal. LCmix
	5%	3.95	1.87	0.08	0.003	0.776	0.215	0.008	0.0006	1.60
	15%	5.76	2.23	0.09	0.004	0.776	0.215	0.008	0.0006	2.17
	35%	8.61	2.70	0.10	0.007	0.776	0.215	0.008	0.0006	2.95
	60%	12.78	3.26	0.12	0.010	0.776	0.215	0.008	0.0006	3.94
	90%	24.13	4.42	0.15	0.020	0.776	0.215	0.008	0.0006	6.04

### Bioassay of binary and quaternary pesticide mixture

Tables 9 and 10 show the test concentrations and the percentage mortality recorded during the pesticide mixture bioassay.

**Table 9:** Observed percentage mortality for *C. gariepinus*.

Pesticide mixture	Concentration (mg/L)	Observed percentage mortality			
		24h	48h	72h	96h
Atrazine-mancozeb	4.61	0	15	30	35
	9.22	10	45	65	65
	13.83	15	55	70	70
	18.44	45	85	90	100
	23.05	70	95	100	100
Atrazine-chlorpyrifos	1.99	20	25	30	65
	3.98	60	60	65	95
	5.97	65	80	95	100
	7.96	90	95	100	100
	9.95	100	100	100	100
Atrazine-lambda cyhalothrin	7.6	5	10	25	45
	10.4	10	20	45	55
	13	30	35	55	60
	15.6	40	45	60	75
	18.2	45	55	70	80
	20.8	85	95	100	100
Mancozeb-chlorpyrifos	0.85	5	5	5	5
	1.11	30	30	35	40
	1.14	35	50	60	65
	1.7	90	90	90	90
	2.26	100	100	100	100
Mancozeb-lambda cyhalothrin	4.33	10	20	25	25
	5.05	25	25	35	40
	5.78	35	35	45	55
	6.5	40	40	55	60
	7.22	55	55	65	75
Chlorpyrifos-lambda cyhalothrin	0.11	10	15	20	25
	0.14	25	25	35	35
	0.18	45	55	55	60
	0.21	60	65	75	80
	0.25	80	85	100	100
Quaternary mixture	0.84	0	5	10	10
	1	15	25	35	40
	1.77	40	45	55	65
	1.34	55	60	75	80
	1.51	100	100	100	100

**Table 10:** Observed percentage mortality for *O. niloticus*.

Pesticide mixture	Concentration	Observed percentage mortality			
		24h	48h	48h	96h
Atrazine-Mancozeb	9.56	0	5	5	10
	10.76	15	35	35	45
	11.95	35	50	65	70
	13.15	45	65	75	85
	14.34	75	90	100	100
Atrazine-Chlorpyrifos	6.99	0	5	5	10
	8.39	10	15	20	25
	9.79	35	40	55	65
	11.18	45	55	65	80
	12.58	55	70	85	95
Atrazine-Lambda cyhalothrin	16.12	0	0	5	10
	22.57	5	5	10	25
	29.02	10	20	35	45
	35.47	25	30	45	65
	41.91	55	75	85	100
Mancozeb-Chlorpyrifos	3.87	0	5	10	10
	4.84	10	15	20	35
	5.81	30	35	45	55
	6.78	55	60	65	80
	7.74	95	100	100	100
Mancozeb-Lambda cyhalothrin	4.5	5	10	10	10
	6.74	20	25	35	40
	8.99	35	45	55	60
	11.24	55	70	75	80
	13.49	80	95	100	100
Chlorpyrifos-Lambda cyhalothrin	0.19	10	20	25	25
	0.29	40	40	55	55
	0.38	60	65	70	75
	0.48	80	80	85	85
	0.57	100	100	100	100
Quaternary Mixture	1.95	5	5	10	10
	3.91	25	30	35	35
	5.86	40	45	55	60
	7.81	70	75	75	80
	11.72	100	100	100	100

**3.2.3 Predicted and Experimental LC_50_ of the pesticide mixture**Table 11 compares the pesticide mixtures' predicted and experimental 96 hr LC_50_. As indicated by the MDR, the hyperbolic model's predictive capability was slightly better than the concentration addition model. MDR equal to 1 indicates perfect compliance between the predicted and observed toxicity of the mixture. MDR greater than1 suggests that the mixture is more toxic than predicted (i.e., an underestimation of mixture toxicity by the models), and MDR less than 1 means that the mixture is less harmful than expected (i.e., an overestimation of mixture toxicity by the models).

**Table 11:** Experimental and predicted 96 hr LC_50_ of the pesticide mixture.

Pesticide Mixture	Mode	Catfish LC_50_ (95% C.I) (mg/l)	Model deviation ratio (MDR)	Nile tilapia LC_50_ (95% C.I) (mg/l)	Model deviation ratio (MDR)
Atrazine-Mancozeb	Observed	11.1 (10.6–11.6)	–	6.7 (2.4–9.7)	–
	HM Predicted	14.3 (12.3–16.3)	1.28	5.9 (4.5–7.1)	0.88
	CA Predicted	18.5 (15.8–21.9)	1.67	6.9 (5.9–8.1)	1.03
Atrazine-Chlorpyrifos	Observed	9.2 (8.6–9.8)	-	2.6 (1.8–3.1)	–
	HM Predicted	8.5 (7.1–9.9)	0.92	4.8 (3.7–5.7)	1.85
	CA Predicted	8.3 (7.1–9.9)	0.90	5.6 (5.1–6.4)	2.15
Atrazine-Lambda cyhalothrin	Observed	22.1 (20.1–38.5)	-	9.3 (6.5–11.1)	–
	HM Predicted	8.7 (7.3–10.1)	0.39	4.51 (3.5–5.4)	0.49
	CA Predicted	8.3 (7.3–9.6)	0.38	5.3 (4.4–6.6)	0.57
Mancozeb-Chlorpyrifos	Observed	5.3 (4.9–5.8)	-	1.2 (1.1–1.3)	–
	HM Predicted	9.7 (8.4–11.7)	1.83	2.5 (2.3–2.8)	2.08
	CA Predicted	11.3 (9.4–13.9)	2.13	1.487^a^	1.24
Mancozeb-Lambda cyhalothrin	Observed	7.5 (6.6–8.5)	-	5.7 (4.9–6.5)	–
	HM Predicted	9.5 (7.8–11.2)	1.27	2.4 (2.1–2.7)	0.42
	CA Predicted	11.3 (9.6–13.5)	1.51	1.6 (1.0–5.0)	0.28
Chlorpyrifos-Lambda cyhalothrin	Observed	0.27 (0.22- 0.31)	-	0.15 (0.13–0.17)	–
	HM Predicted	0.55 (0.467–0.640)	2.04	0.16 (0.15–0.18)	1.07
	CA Predicted	0.24 (0.21–0.28)	0.89	0.06 (0.05–0.07)	0.40
Quaternary Mixture	Observed	4.6 (3.8–5.5)	-	1.09 (1.01–1.17)	–
	HM Predicted	14.9 (12.9–17.1)	3.24	6.0 (4.8–7.0)	5.50
	CA Predicted	9.8 (8.4–11.7)	2.13	3.4 (3.0–4.0)	3.12

a 95% confidence interval could not be estimated. Validity criteria 0.5<MDR<2.

## Discussion

Toxicants acting singly or jointly are categorised as highly toxic if the LC_50_ is between 0.1 and 1 mg/L, moderately toxic if it is between 1 and 10 mg/L, and slightly toxic if it is between 10 and 100 mg/L [14]. The observed LC_50,_ in agreement with the LC_50_ derived from both models indicates that the equitoxic ratio of atrazine-mancozeb was slightly toxic to *C. gariepinus* but moderately toxic to *O. niloticus*. Atrazine-chlorpyrifos was moderately toxic, while chlorpyrifos-lambda cyhalothrin was highly toxic to both species though the LC_50_ data suggests *O niloticus* was more sensitive than *C. gariepinus* to the mixtures. The hyperbolic model also correctly classifies mancozeb-chlorpyrifos and mancozeb-lambda as moderately toxic to both species, though the CA model classed them as slightly toxic. Both models incorrectly categorised the atrazine-lambda toxicity to *C. gariepinus* as moderately rather than slightly toxic. The hyperbolic model differed in classifying quaternary mixture toxic to *C. gariepinus*.

The predicted values by the hyperbolic model may deviate from the observed results if the pesticides in the mixture interact with each other. The term interaction includes all forms of joint action that vary from effect addition, i.e., a more significant effect (synergistic, potentiating) or a lesser effect (antagonistic). Interaction affects the median lethal time of the mixture (a key variable for the hyperbolic model). A previous study showed that synergism decreases time-to-death and lethal concentration, while antagonism increases time-to-death and lethal concentration [9]. The interaction may be toxicokinetic (TK) or toxicodynamic (TD). TK interactions can occur during which a toxicant alters the effective concentration of another, while TD interactions arise when a toxicant influences the organism's response to another toxicant [15].

The predictive accuracy of the CA model may be affected by the different modes of action (MOA) of the pesticides [16]. The concentration addition model is better suited for mixtures whose components share a similar mode of action [17]. This criterion may explain why it performed better than the hyperbolic model in predicting the chlorpyrifos-lambda-cyhalothrin mix, given that both insecticides act on the central nervous system. Wang et al. [18] opined that deviation of the mixture toxicity predicted by the CA model would occur when the components have significantly different slopes.

The deviations within the statistical range of the MDR variation are acceptable and indicate that the observed values were within a factor of 2 of the predicted values [13,16]. MDRs outside this range may provide additional information about a mixture. Cedergreen [16] earlier reported that MDRs may identify mixtures and species groups involved in synergistic (MDR>2), additive (0.5≤MDR≤2), and antagonistic (MDR<0.5) interaction. Synergism may also be present in mixes with MDRs slightly below two [16]. It follows that most pesticide mixtures in this study did not interact but produced an additive effect. The exceptions were antagonism in catfish exposed to atrazine-lambda-cyhalothrin and tilapia exposed to mancozeb-lambda-cyhalothrin, estimated by both models; same with atrazine-lambda-cyhalothrin in Nile tilapia and chlorpyrifos-lambda-cyhalothrin in catfish, estimated by the hyperbolic and CA model, respectively. Furthermore, the MDR suggests a synergistic interaction for the quaternary mixture in both species by both models; same with atrazine-chlorpyrifos in Nile tilapia (CA model), mancozeb-chlorpyrifos in catfish (CA model) and Nile tilapia (HM model), and chlorpyrifos-lambda-cyhalothrin in catfish (HM model).

Some of the predictions by the MDR values do not agree with the results of an earlier study that used the relative toxic unit (RTU), synergistic ratio, and survival analysis to evaluate the interaction of the same pesticide mixture in *O. niloticus* [9]. Atrazine-mancozeb, atrazine-lambda-cyhalothrin, and mancozeb-chlorpyrifos which were synergistic in *O. niloticus* going by the (RTU), synergistic ratio, and survival analysis is not in agreement with the MDRs of both HM and CA model which indicates the toxicity of the mixtures were additive. However the MDR of both models agrees with the other procedure in predicting the mancozeb-lambda-cyhalothrin as antagonistic. This inconsistency suggests that the use of the MDR to estimate mixture interaction may be limited.

Understanding the impacts of pesticide mixtures is essential for the ecological risk assessment of pesticides since the hazard of an individual chemical may be lower than that of a mixture of chemicals [19]. This study showed how the hyperbolic model could play an important role. Then again, the model may be helpful in the pulse exposure assessment of pesticide mixtures to predict the latent effects of pulse exposure to pesticide mixtures once the LT_50_ of the mixture after pulse exposure is known. The main advantage of using the hyperbolic model is that it can estimate the toxic effects of pulse or continuous exposure to a mixture at any concentration level or time of exposure, whereas the concentration addition only uses LC_50_ values at fixed times of exposure. Other models enable the estimation of the harmful effects of single toxicants in organisms with exposure time and concentration but may require other parameters that are not readily available. For instance, the life expectancy reduction model [20] requires the organism's internal LC_50_, LT_50_, and average normal life expectancy. Similarly, the lethality in the time model [21] requires the internal concentration in the organism, rate constant, and bio-concentration factor to predict the toxicity of single toxicants. Others, like the single or multiple pulses model [22], require data on the mortality at exposure time, mortality rate constant, and recovery time to predict toxicity.

## Conclusion

This study showed that the hyperbolic model could predict the percentage mortality and lethal endpoints (LC_50_) of pesticides or any toxicant mixture in fish with the complimentary lethal time addition model. The model can be used during the risk assessment of pesticide mixture in aquatic ecosystems.

## Funding

This research did not receive any specific grant from funding agencies in the public, commercial, or not-for-profit sectors.

## Declaration of Competing Interest

The authors declare that they have no known competing financial interests or personal relationships that could have appeared to influence the work reported in this paper.

## Data Availability

Data will be made available on request.
